# Clean label starch: production, physicochemical characteristics, and industrial applications

**DOI:** 10.1007/s10068-020-00834-3

**Published:** 2020-11-23

**Authors:** Shinjae Park, Yong-Ro Kim

**Affiliations:** 1 Department of Biosystems and Biomaterials Science and Engineering, Seoul National University, Seoul, 08826 Republic of Korea; 2 Research Institute of Agriculture and Life Sciences, Seoul National University, Seoul, 08826 Republic of Korea; 3 Center for Food and Bioconvergence, Seoul National University, Seoul, 08826 Republic of Korea

**Keywords:** Clean label starch, Modified starch, Physical modification, Enzymatic modification

## Abstract

Recently, health-conscious consumers have a tendency to avoid the use of modified starch in their food products because of reluctance regarding food additives or chemical processes. The present paper considers the characteristics and manufacturing methods of clean label starch, which is free from chemical modification. Clean label starch manufacturing is mainly dependent on starch blending, physical and enzymatic modification methods. Physical modifications include ultrasound, hydrothermal (e.g., heat-moisture treatment and annealing), pre-gelatinization (e.g., drum drying, roll drying, spray cooking, and extrusion cooking), high-pressure (high hydrostatic pressure), and pulsed electric field treatments. These physical processes allow variation of starch properties, such as morphological, thermal, rheological, and pasting properties. Enzyme treatment can change the properties of starch more dramatically. Actual use of clean label starch with such altered properties has occurred in industry and is described here. This review may provide useful information on the current status and future direction of clean label starch in the field of food science.

## Introduction

Recently, “clean label food” has emerged as a global food trend (Cheung et al., 2016; Euromonitor-International, 2016). Notably, the movement to attach a clean label to food products that qualifies the essential requirements, such as the use of natural materials and minimum processing, was launched in the United Kingdom in the 1990s (Baines and Seal, 2012). This new trend reflects the evolution of consumers’ concerns on food ingredients and manufacturing. Current consumers are not simply selecting “healthy foods”. They are carefully examining the ingredients in their foods and considering the impact of the product on the environment (Aschemann-Witzel et al., 2019; Seo et al., 2016). Thus, the concept of the clean label is extended to the entire processing of food.

According to Go Clean Label™, the clean label is a consumer-led movement that demands a return to true food through sincerity and transparency. Food containing natural, familiar, and simple ingredients that are easy to recognize, understand, and pronounce should be free from artificial or synthetic chemicals (Hutt and Sloan, 2015). Therefore, the aspects of “free from additives,” “minimum processes,” and “simple ingredient listing” are essential requirements for food to be given a clean label (Aschemann-Witzel et al., 2019; Busken, 2015; Ingredion, 2014). Indeed, many meticulous consumers buy food products with greater consideration regarding whether a clean label is attached (do Nascimento et al., 2018), rather than with consideration of brand awareness. A survey by the Center for Food Integrity found that 75% of the respondents identified nutrition and ingredient labels for their food; 53% considered clean label products to be much healthier. Notably, 46% of Americans said that the availability of food ingredients written in simple terms directly affects their purchasing decisions, and that they are willing to pay higher prices for these foods (Nielsen, 2018). Accordingly, it appears likely that the market size of clean label ingredient foods will grow considerably; it is expected to reach approximately 47.50 billion dollars by 2023, registering a compound annual growth rate of 6.8% during the forecast period (Research-and-Markets, 2018).

Starch, which is the major dietary source of carbohydrates, is an abundant natural ingredient. It is obtained from roots, seeds, tubers, leaves, and stems (Ellis et al., 1998). Depending on the origin of the starch, granules can vary in characteristics such as shape, size, structure, and chemical composition (Smith, 2001). Starch consists of a number of glucose molecules joined together by glycosidic bonds (i.e., amylose and amylopectin) (Ismail et al., 2013; Tharanathan, 2005). Amylose is a linear polysaccharide with α-1,4-glycosidic bonds, which typically constitutes 15–20% (0–70% in particular cases) of starch. Amylopectin, which is a larger branched polysaccharide with α-1,4 and α-1,6 linkages, is a major component of starch (Sajilata et al., 2006; Tharanathan, 2002). In addition to its nutritional aspects, starch has various physical properties based on its composition (e.g., swelling, gelatinization, and gelation); thus, it is used widely in the food industry as a viscosity/freeze–thaw agent and emulsifying stabilizer (Karam et al., 2005). These properties are important for giving food functionality and improving its texture and taste (Chen et al., 2003; Ellis et al., 1998; Jobling, 2004).

However, native starches have limitations in terms of industrial applications, due to their thermal and shear instability and the tendency to retrograde during cooling or freezing, which reduces food product quality (Arocas et al., 2009; Karam et al., 2005; Kokini et al., 1995). Therefore, various studies of modified starch have been conducted, using natural starch altered with small amounts of chemicals to overcome its limitations (Ellis et al., 1998; Zia-ud-Din et al., 2017). The market size of modified starch is also expected to grow from 10.35 billion dollars worldwide in 2018 to 12.67 billion dollars in 2023. Increased consumption of processed and instant foods is the main growth factor in this market (Global-Forecast-to-2023, 2018). Nevertheless, most consumers were also reluctant to buy food with ingredients that were confusing or difficult to understand, as mentioned above. The chemically modified starches are considered as food additives with the label of “modified starch” and E-number coded according to the International Numbering System (lNS). In contrast, physically or enzymatically modified starches as well as native starches are classified as ingredients, not additives. Therefore, they can be labeled simply as “starch” of various sources without E-numbers (Radeloff, 2016). Increasing number of starch manufacturers are producing these clean label starches with a variety of properties similar to those of chemically modified starch in response to consumer trends (Arocas et al., 2009). However, physically modified starches constitute only 5% of the total food-modified starches worldwide and need to be further studied and developed (Radeloff, 2016).

Therefore, this review provides an overview of the current understanding of “clean label starch”. Starch blending and physical modification methods such as ultrasound, hydrothermal, and other treatments are introduced as major ways of manufacturing clean label starch. Enzymatic modification is also presented as an important manufacturing method.

## Starch blending

To counteract the disadvantages of natural starch properties, a small amount of gum (hydrocolloid) is typically added to the product (Oh et al., 2010). However, such hydrocolloids are considered as “additives”, rather than “ingredients”, thus it can be inappropriate for clean label. One of possible alternatives to overcome this involves starch blending. Because starch blending is economical compared to gum addition and is the easiest method to modify the texture of starch gel (Oh et al., 2010; Sun, 2014), many studies have been conducted regarding starch blending. When additive effects are observed in starch blending, the properties of the blend can be predicted through the individual starches involved. However, when these predictions are inconsistent with actual characteristics, non-additive effects are presumed to occur. In such instances, different types of starch characteristics lead to particular interactions in the blend (Waterschoot et al., 2015). In particular, many experiments have shown that the properties of starch blends are related to several factors, including total starch concentration, amylose content, amylose leaching, swelling power, and relative granule size.

### Factors affecting starch blending

#### Starch concentration

Liu and Lelievre (1992) found that the thermal characteristics of a blend were simply the sum of the characteristics of the components when the total starch concentration of the suspension was relatively low (less than 30 wt%). However, non-additive behavior occurs at high starch concentrations because there is competition for water among the blended starch components when the water content is reduced. Waterschoot et al. (2015) also found that each starch is gelatinized independently in the presence of sufficient water. However, in insufficient water, the starch with the lower gelatinization temperature gelatinizes first, leaving less water for gelatinization of the other starch. As a result, gelatinization of the other starch occurs at high temperatures and its characteristics are changed.

#### Relative granule size

Puncha-arnon et al. (2008) reported that blends of canna (mean granule diameter ø = 52.29 ± 0.03 μm) and potato (ø = 47.94 ± 0.20 μm) starches had setback values that were the sum of the contributions from the two starches, whereas the setback values of canna-mung bean (ø = 24.10 ± 0.41 μm) and canna-rice (*ø* = 6.78 ± 1.68 μm) starch blends markedly deviated from the sum of the contributions from each type of starch. These findings indicate that the non-additive effect is more obvious when a larger difference in granule size is present between the starch components of the mixture.

#### Amylose leaching capacity and swelling power

Obanni and Bemiller (1997) found that amylose leaching from starch granules occurs very early in the heating process, before gelatinization or pasting occurs. The leaching of these amyloses from one starch to the surface of other starch can create new molecular interactions and change the pasting properties. Similar results have been obtained in other studies (Gunaratne and Corke, 2007; Zhu and Corke, 2011). Chen et al. (2003) studied the impact of swelling power on pasting properties of a blended sample. They observed that when restricted-swelling starches were mixed with easily swelling starches at a comparable ratio, swelling power was significantly reduced and gelatinization temperature was increased. This occurred because the extent of granular interactions is highly dependent on granular overlap after granule–granule contact, and thus dependent on swelling power.

#### Amylose content

There has been considerable research regarding the characteristic variation of blended starch due to differences in amylose content between components. Novelo-Cen and Betancur-Ancona (2005) researched the chemical and functional properties of *Phaseolus lunatus* (lima bean) and *Manihot esculenta* (cassava) starch blends. The amylose contents of cassava and lima bean were 17.3% and 32.4%, respectively; a specific ratio (lima bean:cassava = 25:75) was determined to provide the best combination of characteristics because of its high viscosity (102 mPa s) without retrogradation. It was also stable in the heating–cooling cycle. These results indicated that new starches with functional properties suitable for use in various foods can be created by mixing native starches from different plant species. Juhász and Salgó (2008) elucidated the pasting properties of corn starch blends by different amylose content: normal corn (27%), waxy corn (0%), and high-amylose corn (70%). Rapid viscosity analysis of system I (mixtures of regular maize starch and high-amylose maize starch or waxy maize starch) and system II (“model” mixtures of waxy maize starch and high-amylose maize starch) showed similar trends, suggesting that functional alteration was highly related to amylose content. However, differences in rheological behaviors of the two systems indicated that starch slurries would be affected by amylose content and interactions between components, or by granule properties (Juhász and Salgó, 2008).

#### Blending with specific starch

Mixing with starch that has unusual properties, such as potato starch, also can be considered as a starch blending technique for manufacturing clean label starches. Potato starch contains phosphorus in the amylopectin portion and its granule size is much larger than that of other starch, which confers distinct properties on potato starch (e.g., high swelling power and metal ion binding capacity) (Tomasik, 2009). Park et al. (2009) investigated pasting profiles of starch mixtures of potato starch and waxy corn starch. They revealed that linear changes occurred in peak viscosity and pasting temperature (additive), but not in breakdown and setback (non-additive), depending on the mixture ratio of the two starches. Sandhu and Kaur (2010) discovered that the addition of potato starch to rice starch substantially affected the characteristics of noodles. Among starch blends, the blending of potato starch and rice starch at the specific ratio of 1:1 resulted in high-quality noodles in terms of shorter cooking time, transparency, and slippery texture. The results revealed the possibility of blending potato starch with rice starch at the same ratio to yield high-quality noodles. Studies of starch blending mentioned thus far (i.e., the types and ratios of starch, as well as the experiments performed) are listed in Table 1.

**Table 1:** Starch blending conditions and examined properties for clean label starch production

Starches	Ratio	Properties	References
Normal corn/waxy corn/high amylose corn/potato/wheat/rice	90:10/80:20/70:30/ 60:40/50:50/40:60/ 30:70/20:80/10:90	Pasting profile/thermal profile/paste morphology	Obanni and Bemiller (1997)
Wheat, rice	100:0/85:15/75:25/ 50:50/25:75/15:85/ 0:100	Thermal profile	Liu and Lelievre (1992)
Rice (indica type/waxy type)		Swelling power/pasting profile/ Thermal profile/granular morphology	Chen et al. (2003)
Corn/cassava/yam	100:0:0/0:100:0/33:34:33/ 50:50:0/20:20:60/67:0:33/ 0:67:33/0:40:60/40:0:60	Gel morphology Texture	Karam et al. (2005)
Lima bean/cassava	100:0/75:25/50:50/ 25:75/0:100	Thermal profile/gel profile/ Pasting profile/texture	Novelo-Cen and Betancur-Ancona (2005)
Canna/potato/mung bean/rice		Pasting profile/thermal profile/ Gel morphology/texture	Puncha-arnon et al. (2008)
Corn/waxy corn/high amylose corn	96:4/83:17/66:33/ 50:50/33:66/17:83/ 4:96	Pasting profile	Juhász and Salgó (2008)
Potato/waxy corn	90:10/80:20/70:30/ 60:40/50:50/40:60/ 30:70/20:80/10:90	Pasting profile/granular morphology	Park et al. (2009)
Rice (flour) with sweet potato/potato/ Cassava/waxy corn/hydroxypropylated potato/hydroxypropylated cassava	2:1 (rice:others)	Rheological profile	Oh et al. (2010)
Rice/potato	75:25/50:50/25:75	Pasting profile/texture	Sandhu and Kaur (2010)
Wheat/sweet potato		Pasting profile/thermal profile/ Texture	Zhu and Corke (2011)

### Applications of starch blending

Blending techniques are mainly used in food industry, such as for noodles (Noda et al., 2006), cakes (Gómez et al., 2008), bread (Koca and Anil, 2007), wheat replacement (Oladunmoye et al., 2014), and gluten reduction (Demirkesen et al., 2010; Mancebo et al., 2016; Paucean et al., 2016).

Noda et al. (2006) investigated the properties of wheat flour potato starch blends for making Chinese instant noodles. The research outcomes revealed that when wheat flour and potato starch blends were used, phosphate presumed to affect the viscosity of potato starch paste was important for making instant noodles with a favorable texture (Noda et al., 2006). Koca and Anil (2007) blended wheat flour with flaxseed (a functional raw material) and studied the rheological and baking properties of flaxseed–wheat blend. When blended with wheat flour up to a ratio of 1:4, flaxseed yielded high-quality bread in terms of loaf volume; moreover, it had no detrimental effect on the sensory characteristics of the bread (Koca and Anil, 2007). Starch blending techniques are especially useful for gluten-free food. Gluten can be difficult for some people to digest, and is presumed to aggravate or cause some health problems (Holmes et al., 1989). Therefore, the reduction of gluten in food is very important. Rice flour (Mancebo et al., 2016; Paucean et al., 2016), chestnut flour (Demirkesen et al., 2010), buckwheat flour (Hadnađev et al., 2013), amaranth (de la Barca et al., 2010), and others have been used to manufacture gluten-free food in numerous studies. Most studies have shown that the texture and appearance of bakery foods with reduced gluten are similar to those of controls.

## Ultrasound treatment

Ultrasound (or ultrasonication) involves the use of sound waves with a frequency higher than the threshold of human hearing (> 20 kHz) (Franco and Bartoli, 2019; Majid et al., 2015). Ultrasound treatment has the potential to produce strong shear force (mechanical), high temperature (physical), and free radicals (chemical) (Mason and Joyce, 2008). Ultrasound treatment is considered as a technology designed to minimize processing, improve quality, and protect food safety (Choi and Lee, 2017; Knorr et al., 2011).

In a starch–water system, ultrasound can change the structure and properties of starch. Because the acoustic energy of ultrasound cannot be absorbed by molecules, it is transformed into a chemically usable form and cause cavitation (Kardos and Luche, 2001; Zhu, 2015). Cavitation induces shear forces that break polymer chains and damage starch granules (Herceg et al., 2010; Jambrak et al., 2010; Zuo et al., 2012). The extent to which starch changes depends on the frequency and intensity of ultrasound, treatment duration, temperature, and the characteristics of starch (e.g., crystallinity and amylose content) (Zhu, 2015). In this section, the effects of ultrasound treatment on starch are described in terms of the various properties changed.

### Characteristics of ultrasound-treated starch

#### Starch morphology

After ultrasound treatment, pits can be observed as black dots on granule surfaces by light microscopy. Zuo et al. (2012) showed that the extent of damage to starch granules depended on sonication power; the increase in defects was found to be linearly related to sonication power. Starch with larger granule size (e.g., potato starch) was found to be more intensely affected by ultrasound treatment, and the extent of erosion was found to increase with longer treatment time (Carmona-García et al., 2016). In addition, more severe granule breaks were observed in areas close to the Maltese cross, corresponding to its fragile surface (Bai et al., 2017; Carmona-García et al., 2016).

Degrois et al. (1974) and Gallant et al. (1972) examined the effects of surrounding gases (e.g., air, hydrogen, oxygen, and carbon dioxide) on the erosion of starch granules during ultrasound treatment. They found that the surface of the starch was roughened and crumpled in air, whereas less damage occurred in oxygen. No damage occurred in carbon dioxide or vacuum. In hydrogen, the pits were large and deep, while the surface was quite smooth.

#### Swelling power and solubility

An increase in the extent of surface fractures can lead to enhanced solubility and swelling power (Carmona-García et al., 2016; Herceg et al., 2010; Zheng et al., 2013). Ultrasound breaks the internal molecular structure and the granular arrangement becomes less compact; therefore, swelling power and solubility increase (Luo et al., 2008). Sujka and Jamroz (2013) experimented with ultrasound treatment on potato, wheat, corn, and using water and ethanol as solvents. The measured solubility and swelling power of all starches increased as a result of the treatment; however, larger increases were observed for granules sonicated in water, compared to those sonicated in ethanol, because depolymerization of the starch occurred to a greater extent when the starch was treated in water.

#### Pasting properties

Ultrasound treatment has been shown to cause collapse of starch granules by cavitation forces and make those granules more permeable to water during the heating step (Herceg et al., 2010). Zheng et al. (2013) investigated the effects of ultrasound frequencies of 25 and 80 kHz on sweet potato starch. They found that the peak viscosities of starch treated with these frequencies were 6.94% and 9.84% lower than those of the native starch, respectively. Generally, viscosity of starch paste is reported to be reduced after ultrasound treatment because partial cleavage of glycosidic linkages occurs, which results in weakening of the polymer network (Herceg et al., 2010; Jambrak et al., 2010; Sujka and Jamroz, 2013). However, some studies have produced the opposite result. Chan et al. (2010) and Carmona-García et al. (2016) found that ultrasound treatment of starch increased the peak viscosity; a larger increase was observed with longer treatment time. The increased in peak viscosity could be attributed to additional treatment time, continuous cycles, and higher amplitudes, all of which allowed the starch granules to decompose and thus absorb more water. Consequently, the granules swelled more and the starch paste became more viscous (Sit et al., 2014). In addition, when starch was subjected to a long period of ultrasound (> 50 min), breakdown substantially increased by the end of treatment, presumably due to the fragility of starch granules after treatment (Carmona-García et al., 2016). Therefore, the effect of ultrasound treatment on pasting properties of starch depended on the treatment conditions.

#### Retrogradation properties

Retrogradation properties have not been extensively affected by low ultrasound power and intensity. However, high ultrasound power and strong intensity effectively increased the onset temperature and reduced the enthalpy of rice starch, thereby reducing the retrogradation enthalpy (Yu et al., 2013). Nie et al. (2019) investigated the effects of ultrasound treatment on the retrogradation of potato starch paste. They discovered that ultrasound treatment changed the crystallinity type of potato starch from type B to type V. The results indicated that ultrasound caused the formation of a V-type structure, which facilitates water absorption; thus, the crystal structure and crystallinity of retrograded potato starch were greatly affected by ultrasound treatment (Nie et al., 2019).

### Applications of ultrasound-treated starch

Ultrasound-treated starch has been widely used in various food-related applications in the laboratory and the commercial setting (Zhu, 2015). Examples include an enzymatic modification enhancer (Wu et al., 2011), encapsulation and delivery system (Xing et al., 2014; Zhu, 2017), emulsifier (Abbas et al., 2014), and edible film (Cheng et al., 2010), as well as use in V-type inclusion complex formation (Tian et al., 2013) and bioethanol production. (Khanal et al., 2007; Nitayavardhana et al., 2010).

Wu et al. (2011) studied acceleration of pore formation and enhancement of the specific surface area of microporous starch granules by using the combination of glucoamylase and ultrasound treatment. Among the treatment groups for microporous starch granule preparation, the method involving ultrasound treatment with simultaneous glucoamylase digestion was the most effective (Wu et al., 2011). Xing et al. (2014) elucidated the encapsulation efficacy of *Lactobacillus acidophilus* using ultrasound-treated porous starch, and the survival rate of *L. acidophilus* in gastrointestinal fluid was the highest when it was encapsulated with a 10% concentration of porous starch. Ultrasound treatment also affected the emulsifying ability of octenyl succinic anhydride (OSA) starch. Abbas et al. (2014) found that when OSA starch was subjected to ultrasound treatment under certain conditions (power density: 1.45 W mL^−1^, time: 7 min), the maximum amount of curcumin could be loaded into medium-chain triacylglycerols while reducing the emulsifier concentration. Additionally, Cheng et al. (2010) used the modified properties (good transparency, improved moisture resistance, and stronger structure) of starch after ultrasound treatment to produce edible films. Khanal et al. (2007) and Nitayavardhana et al. (2010) used ultrasound-treated corn/cassava starch to produce bioethanol, and notably, the duration of ethanol fermentation was reduced by almost 24 h in the ultrasound-pretreated group, compared to the control group.

## Hydrothermal treatment

Heat–moisture treatment (HMT) and annealing (ANN) are two common types of hydrothermal treatment that change the physicochemical properties of starch without rupturing its granular structure (Adebowale et al., 2005; Jacobs and Delcour, 1998; Zavareze and Dias, 2011). Typically, HMT is conducted by heating starch with limited moisture contents (10–40%, w/w) at temperatures (usually above 94 °C) above its glass transition temperature that depends on the moisture level during 1 to sometimes more than 24 h (Chung et al., 2009). Differently from HMT, ANN is performed in an excess of water (> 60%) at a temperature above glass transitions temperature and below the gelatinization temperature of starch (generally 5–15 °C below the gelatinization onset temperature) (Zavareze and Dias, 2011).

Generally, increases in gelatinization temperature, thermal stability, and crystallinity (Abraham, 1993), diminished granular swelling due to reduction of amylose leaching, strengthened intramolecular bonds (Jiranuntakul et al., 2011), and widening of the gelatinization temperature range have been demonstrated after HMT regardless of starch sources (Chung et al., 2009). ANN has shown to induce elevated granule stability, high gelatinization temperatures, crystalline perfection, and a greater number of starch chain interactions. It has also shown to reduce amylose leaching and granular swelling. Notably, the results varied depending on the source of the starch, formation of the amylose–lipid complex, decay of crystallinity, and range of enzyme susceptibility (Chung et al., 2009). Typical changes provoked by ANN and HMT are shown in Table 2.

**Table 2:** Characteristic change by heat-moisture treatment (HMT) and annealing (ANN) (in general)

Properties	HMT	ANN	References
Swelling power	−	−	Abraham (1993), Adebowale et al. (2005), Chung et al. (2000), Chung et al. (2009), Gomes et al. (2004), Gomes et al. (2005), Jacobs and Delcour (1998), Puncha-arnon and Uttapap (2013), Song et al. (2011), Watcharatewinkul et al. (2009), Zavareze and Dias (2011)
Solubility	−	−	
Amylose leaching	−	−	
Gelatinization temp.	+	+	
Peak viscosity	−	+/−	
Breakdown	−	−	
Setback	+	+/−	
Gel hardness	++	+	
Crystallinity	+	+	
SDS/RS	++	+	

+, increased; −, decreased

### Characteristics of HMT/ANN starch

#### Pasting properties

HMT and ANN promote significant changes in starch pasting properties. In starch subjected to HMT, a reduction in breakdown has been demonstrated, indicating that starches becomes more stable during continuous heating and shearing (Abraham, 1993; Jiranuntakul et al., 2011; Puncha-arnon and Uttapap, 2013; Watcharatewinkul et al., 2009). Puncha-arnon and Uttapap (2013) studied the changes in starch properties after HMT depending on water content. The pasting temperature of HMT starch with 20% moisture content slightly increased, but a significant increase was observed for 25% and 30% moisture contents HMT starches. Watcharatewinkul et al. (2009) noted that HMT of canna starch resulted in lower viscosity, higher paste stability, and elevated gelatinization temperature, indicating that it partly demonstrated the properties of cross-linked starch.

Compared to HMT, the pasting properties attained by ANN vary depending on experimental conditions (Zavareze and Dias, 2011). Adebowale et al. (2005) claimed that annealed sorghum starch showed higher peak viscosity than native and heat–moisture treated starch due to unrestricted swelling of the starch. Jacobs et al. (1996) also claimed that ANN increased the peak and final viscosities of wheat and rice starches. However, Gomes et al. (2005) and Singh et al. (2011) reported that ANN, like HMT, lowered the peak viscosity/swelling power of cassava and sorghum, respectively. Gomes et al. (2004) reported that cassava starch attained low peak viscosity and low breakdown through ANN, but the final viscosity was elevated, compared to the control.

#### Gel texture and rheological properties

Both HMT and ANN approaches resulted in greater gel hardness due to changes in the gel network, such as enhanced amylose cross-linking/crystalline perfection/higher gel density caused by reduced swelling power (Cham and Suwannaporn, 2010; Eerlingen et al., 1997; Jacobs and Delcour, 1998; Liu et al., 2000). Eerlingen et al. (1997) studied the rheological properties of potato starch treated under various moisture levels (20–40%) and at temperatures few degree below the peak gelatinization temperature. They found that HMT led to reduced amylose leaching, solubility, and swelling power, whereas it led to an enhanced close packing concentration, which increased the storage modulus (G’) of the gel. Collado and Corke (1999) used HMT to treat sweet potato starch with varying amylose content and examined the gel texture. They reported that “Taiwan starch” (amylose content: 15.2%) gel had marked enhancements in hardness and adhesiveness, whereas “93-006” (amylose content: 28.5%) did not show significant differences in hardness. These findings indicate that the amylose content of native starch has a considerable effect on HMT starch gel.

Cham and Suwannaporn (2010) examined the physical properties of noodle produced with physically modified starch. They found that the G’ after cooling of HMT was much higher than that of ANN. Jacobs and Delcour (1998) revealed that the elastic moduli of gels made of annealed wheat starches were higher than those of the starting materials, concluding that ANN treatment increased the strength of the gels. Chung et al. (2000) also reported that ANN caused rearrangement of starch molecules, resulting in reduced solubility and swelling power and enhanced gel strength.

#### Slowly digestible starch/resistant starch

Starch is generally classified as rapidly digestible starch (RDS), slowly digestible starch (SDS), and resistant starch (RS), depending on the digestion rate (Juansang et al., 2012). In several studies, HMT and ANN have been used to make SDS and RS (Chung et al., 2009; Song et al., 2011). Chung et al. (2009) conducted ANN and HMT with corn, soy, and lentil starch, and measured the amount of SDS/RS and glycemic index of each starch according to digestion rate. Notably, they found that susceptibility of each starch to enzyme digestion was substantially affected by HMT and ANN, which produced SDS and RS with high thermal stability. The increase in RS after ANN and HMT reflected enhanced interactions between starch chains and crystalline perfection. Song et al. (2011) found that non-waxy rice treated with ANN exhibited properties similar to those of cross-linked starch, due to rearrangement of the outer branch of amylopectin in rice starch granules, which reduced the gap between starch molecules.

### Applications of HMT/ANN starch

Hydrothermally treated starches are available for use in the canned and frozen food industries because of their high thermal stability and reduced retrogradation tendency (Jayakody and Hoover, 2008). Additionally, HMT starches could be used in pasta, bread, and noodles (Bourekoua et al., 2016; Cham and Suwannaporn, 2010; Suzuki and Sekiya, 1994). Bourekoua et al. (2016) tested the impact of HMT rice and corn flours on gluten-free bakery goods. They performed HMT of rice flour and corn flour at 65 °C with a powder-to-water ratio of 5:1. The results showed that hydrothermal treatment of rice or corn flour had various effects on the characteristics of bread (e.g., specific volume, height/width ratio, hardness, and chewiness), and that those effects were acceptable in comparison with the control group. Cham and Suwannaporn (2010) were able to produce natural and safer rice noodles with food quality similar to that of noodles from commercial rice flour, by conducting hydrothermal treatment. ANN treatment was appropriate when rice noodles required a softer texture, while HMT was more suitable for production of semi-dry and dried noodles, which required higher tensile strength and gel hardness. In addition, the physically modified rice powder had low amylose content, which enabled production of “clean and smooth” noodles. Through total dietary fiber analysis, Brumovsky and Thompson (2001) showed that partial acid hydrolysis of “Hylon VII” (high-amylose corn starch) for 6 h at 25 °C, followed by ANN (24 h, 70 °C, moisture content: 67%), increased RS content by 32%. This RS exhibited a relatively bland taste, white color, and micro-particulate structure, thus, it could be incorporated into food without changing the food’s appearance and texture. Notably, it has been used as a fat mimetic or to increase the dietary fiber content of food (Jayakody and Hoover, 2008).

## Pre-gelatinization

Pre-gelatinized starch is physically modified starch that has undergone a particular cooking process (complete gelatinization and simultaneous or subsequent drying) (Alcázar-Alay and Meireles, 2015). Pre-gelatinization methods include drum drying, roll drying, spray cooking, and extrusion cooking. These processes cause starch granules to swell irreversibly and dissolve the intermolecular hydrogen bonds of starch molecules (Radeloff, 2016). Because of these changes, pre-gelatinized starch can melt easily in cold water. Furthermore, no heat treatment is needed to manufacture the paste form, making it a suitable method for heat-sensitive foods (Alcázar-Alay and Meireles, 2015).

### Preparation of pre-gelatinized starch

#### Drum and roll drying

Drum drying is a common method in the food industry for pre-cooking grain-based products, such as gruel and porridge (Björck et al., 1984). The process is performed in several steps. First, the starch slurry is applied as a thin layer to the outer surface of the drum as the inside of the drum dryer is heated. One rotation later, the immediately dried starch slurry is obtained in film or flake form by a knife attached to the dryer. It is then milled to a finished flake or powder form (Radeloff, 2016). Valous et al. (2002) used a double drum dryer to pre-gelatinize corn starch and observed some complex interactions among steam pressure, drum rotation speed, and pre-gelatinized pool level during the operation. All input variables had significant effects related to drum temperature and width of the gap between the drums. The moisture content, mass flow rate, and specific load of the dried product decreased as the steam pressure increased. When the drum speed increased, the response of the dryer varied depending on the steam pressure (Valous et al., 2002). Wiriyawattana et al. (2018) investigated the effects of drum drying on the physical and antioxidant properties of pre-gelatinized riceberry flour. They found that the water absorption index and swelling power of all pre-gelatinized riceberry flour samples were significantly higher than samples in the control group. Moreover, the pasting temperature, peak viscosity, trough viscosity, final viscosity, and setback of the riceberry flour were reduced after drum drying (Wiriyawattana et al., 2018).

#### Extrusion cooking

Extrusion cooking is the process of applying mechanical shear force and high temperature to starch granules for a short period of time in a relatively low humidity environment (Radeloff, 2016). This process changes some of the functional properties of starch. Notably, high temperature–short time extrusion cooking is used by many food industries to produce expanded snack foods, ready-to-eat cereals, and pet foods (Chinnaswamy and Hanna, 1988). Parchure and Kulkarni (1997) investigated the RS content in rice and amaranth that had changed due to extrusion cooking. They found that extrusion cooking treatment reduced RS content compared with that of the respective native starches, and this change could be explained by enhanced solubilization of starch due to macromolecular degradation.

#### Spray cooking

Simultaneous starch cooking and spray drying in a chamber maintained at a certain temperature and pressure produces spray-type starch with the characteristics of cold water-swelling starch (Radeloff, 2016). Fu et al. (2012) investigated the physical properties of partially gelatinized corn starch obtained by gelatinization, followed by spray drying. The partially gelatinized starch had a mostly amorphous structure, thus, the swelling power of the partially gelatinized starch was much higher than that of native corn starch at temperatures < 60 °C. This finding indicated that the partially gelatinized starch with spray drying had better hydration at low temperature. Izidoro et al. (2011) investigated the effect of spray drying on ultrasound-treated green banana starch. The RS content was significantly lower when the starch underwent simultaneous spray dry and ultrasound treatment, presumably due to reduction of starch crystallization. The researchers demonstrated that the spray drying technique increased the solubility, swelling power, and water absorption capacity of green banana starch.

### Applications of pre-gelatinized starch

Most pre-gelatinized starch is widely used in a variety of food products. Majzoobi et al. (2011) produced pre-gelatinized starch using the drum dryer and reported the characteristics of the starch produced, those were low crystallinity, high cold water viscosity, high water solubility, and high absorption. Based on these properties, they suggested that pre-gelatinized starch could be used mainly as a thickening and gelling agent in frozen/instant foods or heat-sensitive products, such as cold desserts, salad dressings, bakery mixes, and baby foods (Alcázar-Alay and Meireles, 2015; Majzoobi et al., 2011).

Additionally, in the pharmaceutical field, pre-gelatinization has great potential in producing capsule and tablet diluents, as well as capsule decomposers. Which enable the starch to absorb water so that the pills disintegrate properly (controlled release) (Anwar et al., 2006; Te Wierik et al., 1997). Te Wierik et al. (1997) described the general applicability of retrograded pre-gelatinized starch products in directly compressible controlled-release matrix systems. The rate of release from retrograded pre-gelatinized starch tablets may increase or decrease in relation to the desired profile of other parameters, such as the compression force and geometry of the tablet. These results demonstrated that the release of chemicals can be controlled through pre-gelatinized starch with various characteristics.

## High pressure treatment

The use of high pressure (or high hydrostatic pressure) for food processing and preservation has been investigated as an alternative to traditional treatment (Stolt et al., 2000). High-pressure treatment is a non-thermal modification method, and pressure-induced disordering is similar, but not identical, to heat-induced disordering (Guo et al., 2015; Oh et al., 2008). This process can be considered as an appropriate technique for the production of minimally processed foods. It has potential uses for new products with unique textures or tastes and has minimal effects on flavor, color, or nutritional value. Notably, it does not involve any thermal degradation (Pei-Ling et al., 2010). This technique can also be applied to starch, thereby changing starch structure, retrogradation, and chemical reactions. There are also reported that amorphous granular starches have been produced (Song et al., 2015; Song et al., 2017). The characteristics of starch under high-pressure treatment conditions differ considerably depending on the treatment pressure, pressurization temperature, and pressurization time (Pei-Ling et al., 2010).

### Characteristics of high pressure-treated starch

#### Starch morphology

**Fig. 1:**
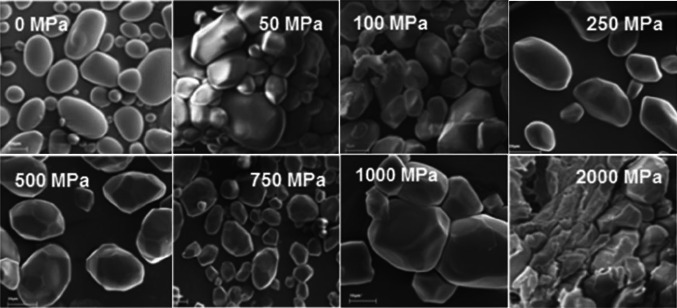
SEM microstructure of potato starch: native and pressurized starches under pressures in the range of 50–2000 MPa (Słomińska et al. 2015)

Stolt et al. (2000) investigated the effect of high-pressure treatment on barley starch and found that the granular structure of the starch was maintained after 50 min of treatment at 600 MPa, while the birefringence nearly completely disappeared after 30 min of retention time at 450 MPa. Błaszczak et al. (2005) conducted high-pressure treatment of potato starch at 600 MPa for 2–3 min and observed its granular structure using SEM. Many granules showed considerable pressure-related deformation. The outside of granules seemed more resistant to change exhibiting a very compact, condensed layer, while the inside of granules exhibiting relatively coarse structure clearly showed destroyed and gel-like structures. The reduction of birefringence and the formation of gel-like structure (result of hydration of the amorphous phase and/or melting of the crystalline structure) indicated that starch became gelatinized under pressure (Pei-Ling et al., 2010). Meanwhile, Song et al. (2015) investigated the possibility of amorphous granular starch (AGS) preparation from several starches with non-thermal high-pressure treatment. They found that corn, tapioca and non-waxy rice starches became AGSs upon specific treatment condition (550 MPa for 30 min). These starches completely gelatinized but maintained granular structures, successfully achieving AGSs. Figure 1 shows the scanning electron microscope microstructure of potato starch, depending on pressure.

#### Pasting properties

The degree of starch gelatinization has been shown to increase with higher pressurization treatment temperature in the temperature range between 25 °C and the heat gelatinization temperature (Muhr and Blanshard, 1982). In addition, when a starch suspension was heated under a pressure of > 200 MPa, starch gelatinization was able to occur even in a markedly lower temperature range (room temperature) (Lee et al., 2006). The degree of gelatinization considerably increased as the pressurization treatment time increases within the range of up to 60 min. Moreover, the consistency index, melting enthalpy, and differential scanning calorimetry peak temperature increased with enhanced pressure and pressurization time (Pei-Ling et al., 2010). However, processing time longer than 60 min did not cause further effect on gelatinization, presumably because the change in starch reached a plateau level with a processing time of > 60 min.

#### Retrogradation properties

The retrogradation of pressure-induced gels should differ from that of heat-induced gels because amylose leaching is rarely observed after pressurization. The rate of retrogradation is generally known to be more rapid in heat-gelatinized starch than in pressure-gelatinized starch (Douzals et al., 1996). Guo et al. (2015) found that high-pressure (100–500 MPa)-treated lotus seed starch had significantly higher pasting temperature, peak viscosity, trough viscosity, and final viscosity values, compared to native starch. However, opposite results were obtained for breakdown and setback. As breakdown indicates the stability and resistance of starch granules to shear stress, while setback represents the rapid retrogradation of leached amylose in starch paste, the high-pressure-treated lotus seed starch had higher granular stability and a lower retrogradation tendency, compared to native starch (Guo et al., 2015). Hu et al. (2011) investigated the retrogradation rate of pressure-gelatinized rice starches, as compared to heat-gelatinized starches, and found that the retrogradation rate of normal rice starch gelatinized by high pressure was lower than that of normal rice starch gelatinized by heat, due to fewer broken granules and lower leached amylose in pressure-gelatinized rice starch.

#### Rheological properties

Ahmed et al. (2018) examined the impact of high-pressure treatment with different pressures and starch/water ratios on the gelatinization properties of quinoa starch. Frequency sweep analysis showed that the pressurized starch suspension had mainly solid-like behavior with *G′* > *G*″ at all selected frequency ranges (0.1–10 Hz). The extent of quinoa starch gelatinization, so thus the conversion of starch suspension to a gel, increased as pressure increased. This trend became greater as both pressure exerted and starch concentration increased. According to many other studies on the effect of high-pressure treatment on the rheological properties of various starches, including those of sorghum (Vallons and Arendt, 2009), mung bean (Jiang et al., 2015a), and rice (Jiang et al., 2015b), the moduli of most starch gels significantly increased as the treatment pressure level increased. In a steady shear flow experiments, the consistency index increased as pressure increased, while the flow behavior index converged to zero (Jiang et al., 2015a). These findings imply that harder gel can be formed without heat treatment by applying higher pressure to starch solution.

### Applications of high pressure-treated starch

Gelatinization at room temperature or even below room temperature after high-pressure treatment can be a great potential to save energy in many food industries. For example, when starch paste at a concentration of > 15% is processed with this technique, the resulting creamy texture paste can replace oil in low-fat foods (e.g., mayonnaise, confectionery foods, desserts, and dairy products) without heat treatment (Nasehi and Javaheri, 2012). High-pressure treatment also produces RS that has roles similar to soluble fiber and its use is recommended for controlling diabetes and colorectal cancer (Nasehi and Javaheri, 2012). In addition to physicochemical and nutritional characteristics, the effect of this technique on the sensory characteristics of final products can also be considered (Pei-Ling et al., 2010).

## Pulsed electric field treatment

Thermal processing is the most common method to kill contaminating microorganisms. However, this method reduces the taste, color, and nutritional value of food. Various non-thermal methods have been developed to overcome these limitations, and the pulsed electric field (PEF) method is one of these approaches (Jeyamkondan et al., 1999). This technology treats pumpable liquid materials in a processing chamber with high-intensity electric pulses (> 10 kV cm^−1^) for short durations (< 40 μs) (Han et al., 2012). The short duration is used to prevent excessive heat and unwanted electrolytic reactions (Zhu, 2018). Relatively recently, Recently, PEF treatment has been used more frequently to manufacture physically modified starch due to its characteristics of low processing temperature, continuous processing nature, short treatment time, and uniform treatment intensity, as well as other aspects (Han et al., 2012; Han et al., 2009b; Li et al., 2019; Zeng et al., 2016). In this section, the effects of PEF on the properties of starch are discussed in terms of developing a useful method to produce clean label starch for industrial applications.

### Characteristics of PEF-treated starch

#### Starch morphology

The surface morphology of examined native starch granules is smooth, and Maltese crosses are clearly observed at low electric field intensity, but damage begins to emerge as the intensity exceeds 30 kV cm^−1^. When the intensity reaches 50 kV cm^−1^, the shape of the starch is nearly destroyed and aggregates of fragments appear to form a gel network (Han et al., 2009b; Han et al., 2012; Li et al., 2019; Zeng et al., 2016). The morphological change suggests that electric energy can act on the inside of starch granules and on outer structures. The outer part of a starch granule is known to have a very dense layer, which increases resistance to external physical stimulation (Błaszczak et al., 2005). After PEF treatment, the interior of starch granules—which have lost their protective outer envelopes—is severely damaged and can absorb water more effectively and swell easily.

#### Crystalline structure

Starches are classified into three types according to their patterns of crystallinity, type A, type B, and type C. Li et al. (2019) examined whether PEF (intensity: 0–8.57 kV cm^−1^) processing could change the structure of wheat starch (type A), potato starch (type B), and pea starch (type C). They used polarized light microscopy, X-ray diffractometry, attenuated total reflectance Fourier transform infrared spectroscopy (ATR-FTIR), solid-state nuclear magnetic resonance spectroscopy, small-angle X-ray scattering, and gel permeation chromatography to identify the effect of PEF on the starch. The results showed that PEF could change the structures of all three types of starch, especially potato starch. Although treatment did not change the X-ray diffraction pattern type, it changed the strength of the peaks in ATR-FTIR, as well as the molecular weight distribution (Li et al., 2019). Overall, it seems that PEF with high electric field strength may cause gelatinization of surfaces and partial disruption of starch crystallinity (Zhu, 2018).

#### Pasting properties

PEF at a high electric field strength (> 50 kV cm^−1^) tends to reduce the peak viscosity, breakdown, and setback value of starch during pasting (Han et al., 2009a; 2009b; Han et al., 2012). Han et al. (2012) studied the effects of PEF (intensity: 30, 40, and 50 kV cm^−1^) in tapioca starch–water dispersion solutions (8.0%, w/w). When the electric field strength was increased from 30 to 50 kV cm^−1^, the peak viscosity of the processed sample gradually decreased, and the difference in peak viscosity between native and processed samples showed a corresponding increase from 61 to 162 BU (Han et al., 2012). Thermal analysis by differential scanning calorimetry has shown that PEF tends to reduce ΔH and gelatinization temperature. The reduced gelatinization temperature also suggests that PEF disrupts the crystalline region (Zhu, 2018).

### Applications of PEF-treated starch

Originally, PEF technology was successfully applied for the sterilization of a variety of liquid foods with low viscosity and low electrical conductivity (e.g., milk, soup, liquid egg, and assorted fruit juices) (Han et al., 2009b; Zeng et al., 2016). Recently, Li et al. (2019) found that when PEF was used with wheat, potato, and pea starches, it could diversify the rate of starch digestion, producing RDS, SDS, and RS. For all starches, RDS content increased significantly depending on the electric field intensity, while SDS content was lower in treated samples than in the control group at all electric field intensities. For RS, different tendencies were displayed for different types of starch. The RS content in wheat and potato starch increased significantly when the starch was treated with low-intensity PEF (2.86 and 4.29 kV cm^−1^). However, the value did not change considerably as the intensity of the electric field increased (5.71, 7.14, and 8.57 kV cm^−1^). The RS of pea starch did not exhibit substantial change at any electric intensity (Li et al., 2019). Based on these results, regulation of the starch digestion rate according to PEF intensity is available to various starch food industries.

## Enzymatic modification

Enzymes are highly selective catalysts, which greatly enhance both the rate and specificity of biochemical and metabolic reactions. Enzymatic modification has several advantages, such as elimination of undesirable byproducts, improved purity, low cost and consistency of high-quality products (Radeloff, 2016). Enzymatic processes are also applied to starch to add some novel functionalities and mimic the properties of chemically modified starch (Patil, 2013; Radeloff, 2016). Notably, starch treated in this manner can be classified as clean label starch because the method does not use chemicals. This section covers the types of enzymes used in enzymatic processes and their applications.

### Enzymes used for clean label starch production

Among various enzymes acting on starch, α-glucanotransferases (αGTases) have been extensively used for clean label starch production. αGTases, which belong to the superfamily of glycoside hydrolases (GHs), cleave the α-1,4-glycosidic bond in the substrate such as amylose, amylopectin, glycogen, and maltodextrins. However, these transferases do not simply split the glycosidic bond. They transfer the removed glucan molecule from the donor to the non-reducing end of other glycosidic acceptor, thus forming a new glycosidic bond. This category includes 4-α-glucanotransferase (4αGTase, EC 2.4.1.25), cyclodextrin glycosyltransferase (CGTase, EC 2.4.1.19), and branching enzyme (BE, EC 2.4.1.18) (van der Maarel et al., 2002).

CGTase can form cyclic oligosaccharides known as cyclodextrin (6, 7, or 8 glucose residues) that have the ability to form inclusion complexes with specific guest molecules (Uekama et al., 1998). 4αGTase also known as amylomaltase and D-enzyme (disproportionationg enzyme), catalyses transglycosylation reaction on the starch substrate through disproportionation, cyclization, coupling and hydrolysis reactions (Takaha et al., 1996). This enzyme can produce a modified amylopectin clusters and cyclic products of α-1,4-glucan called cycloamyloses or large-ring cyclodextrins by inter and intramolecular transglycosylation (Nimpiboon et al., 2020). It is also possible to manufacture thermoreversible starch gel using this enzyme (Kaper et al., 2005; van der Maarel et al., 2005). Branching enzyme, which is responsible for synthesis of α-1,6-glucosidic bonds in starch and glycogen in vivo, cleaves an_α-1,4-glycosidic linkage similarly as above two enzymes, but transfer the cleaved α-glucan chain to a free 6-hydroxyl group in an acceptor glucan chain forming a α-1,6-glycosodic bond called branch (van der Maarel and Leemhuis, 2013). It catalyzes not only the branching reaction but also a cyclization reaction of amylose and amylopectin (Takata et al., 2010).

### Applications of enzymatically modified starch

α GTases has received considerable attention leading to a number of new commercial products over the last 10 years (van der Maarel and Leemhuis, 2013). α GTases-treated starch products include cyclodextrin (Li et al., 2014; Szente and Szejtli, 2004), thermoreversible starch gel (Patil, 2013), slowly digestible starch (SDS) and resistant starch (RS) (Jo et al., 2016), highly branched cyclic dextrin (Takii et al., 2007).

The application of cyclodextrin-assisted molecular encapsulation in foods offers various benefits such as solubility enhancement of water insoluble compounds, protection of active ingredients against the external environment, elimination (or reduction) of undesired components, technological advantages that include simple dosing and handling, economic advantages, and labor savings (Szente and Szejtli, 2004). Therefore, cyclodextrin is widely used in the food industry to improve the solubility and stability of flavors, vitamins, colorants, and unsaturated fats (Szente and Szejtli, 2004). Notably, β-cyclodextrin (DP 7 cyclic oligosaccharides) is the most accessible, the lowest-priced and generally the most useful, thus it is suitable for use in the industry compared to other types of cyclodextrins (Del Valle, 2004). Meanwhile, Li et al. (2014) focused on the unique properties and food applications of α-cyclodextrin (DP 6 cyclic oligosaccharides). Compared to other cyclodextrins, α-cyclodextrin exhibited the smallest internal cavity and highest resistance to enzymatic hydrolysis, thus, it can be used for special applications in food industry, as a natural and soluble dietary fiber (Li et al., 2014). According to Patil (2013), 4αGTase-treated starches are increasingly used as food ingredients. 4αGTase has enzyme reaction pattern very similar to those of CGTase. One of major differences is that 4αGTase produces relatively large molecular weight enzyme products (10^4^–10^5^ Da) and various sizes of large ring cyclic glucans (mainly DP 17–40 from amylose) from starch substrate, whereas CGTase significantly reduces the molecular weight of starch and mainly produces small circular products of DP 6, 7, and 8 (van der Maarel et al., 2002). Therefore, starch treated with 4αGTase exhibits unique thermoreversible gel forming characteristics (Do et al., 2012; Lee et al., 2008). During repeated heating and cooling cycle, it can be melted or hardened several times, as similar to gelatin. 4αGTase-treated starch is available in the market as a trade name of Etenia™, and can be used as an alternative to gelatin particularly in jelly-type confectioneries, for low-fat dairy products of enhanced creaminess and mouth feel, and emulsified low-fat spread (Patil, 2013). Jo et al. (2016) conducted an experiment using glycogen branching enzyme from *Streptococcus mutans* and amylosucrase from *Neisseria polysaccharea* to increase the SDS or RS contents of sweet potato starch. They found that the SDS and RS contents of enzyme-treated starches increased from 6.3 to 25.0%–34.8% and from 12.9 to 34.6%–41.3%, respectively. Increased number of branch points and elongated chain length accelerated retrogradation of amylopectin, and it could bring slow digestion property to the starch. Highly branched cyclic dextrin (HBCD) is synthesized using branching enzyme from *Bacillus stearothermophilus*, and available as a food ingredient with the trade name of cyclic cluster dextrin (CCD) (Takata et al., 2010). The product has been used as a component of sports drinks and a spray-drying aid, and for the reduction of unpleasant flavor and other applications (Takata et al., 2010). Takii et al. (2007) studied the gastric emptying time of HBCD-based sports drink, in comparison with other carbohydrate-based sports drinks during exercise. The mean gastric emptying time after consumption of the HBCD-based sports drink was significantly lower than that of the typical sports drinks. In addition, when volunteers drank a 10% HBCD-based sports drink during bicycle exercise, they experienced few gastrointestinal symptoms and could comfortably continue their exercise with little fatigue (Takii et al., 2007).

In conclusion, this review has provided an overview of the currently available g clean label starch. Clean label starch, made without the use of chemicals, is not expected to completely replace conventional modified starch at the present time, due to various limitations. However, there remain many opportunities for use of clean label starch, because researchers are continuously moving the field forward with new discoveries pertaining to processes, new enzymes, and cost-effective options for consumers. Therefore, although clean label starch currently constitutes only 5% of the global modified starch market, it will increasingly replace modified starch.
